# Replication Stress: A Lifetime of Epigenetic Change

**DOI:** 10.3390/genes6030858

**Published:** 2015-09-11

**Authors:** Simran Khurana, Philipp Oberdoerffer

**Affiliations:** Laboratory for Receptor Biology and Gene Expression, National Cancer Institute, NIH, 41 Library Drive, Bethesda, MD 20892, USA

**Keywords:** replication stress, DNA repair, chromatin, histones, senescence

## Abstract

DNA replication is essential for cell division. Challenges to the progression of DNA polymerase can result in replication stress, promoting the stalling and ultimately collapse of replication forks. The latter involves the formation of DNA double-strand breaks (DSBs) and has been linked to both genome instability and irreversible cell cycle arrest (senescence). Recent technological advances have elucidated many of the factors that contribute to the sensing and repair of stalled or broken replication forks. In addition to *bona fide* repair factors, these efforts highlight a range of chromatin-associated changes at and near sites of replication stress, suggesting defects in epigenome maintenance as a potential outcome of aberrant DNA replication. Here, we will summarize recent insight into replication stress-induced chromatin-reorganization and will speculate on possible adverse effects for gene expression, nuclear integrity and, ultimately, cell function.

## 1. Introduction

The average human body undergoes approximately 10,000 trillion cell divisions over a lifetime. These include asymmetric divisions to generate differentiated cell types from less differentiated stem or progenitor cells and symmetric divisions to expand or maintain a given cell type. Irrespective of the nature of the division, each cell will need to ensure accurate replication of its genetic material. Moreover, epigenetic information needs to be maintained during symmetric cell divisions. These processes are far from error-free, and with the exception of stem cells, an individual cell typically undergoes no more than 40 to 60 divisions before it enters a state of irreversible cell cycle arrest termed senescence. The latter is characterized by significant changes in the epigenome, as well as evidence for persistent DNA damage, suggesting alterations in nuclear integrity as a contributing factor [1,2]. Indeed, senescence is in large part attributed to a critical shortening of the protective ends of chromosomes, a process termed telomere erosion, which causes cumulative DNA damage and, ultimately, cell cycle arrest [3]. Recent findings further suggest the involvement of alternative pathways that are tightly linked to defects in the replication of genomic DNA, and a concomitant, aberrant cellular response to replication stress. Similar to telomere erosion, these cases result in an activation of the DNA damage response (DDR), which can trigger permanent cell cycle arrest [1].

DNA replication, as well as the cellular response to replication stress, occurs in the context of chromatin, a protein/DNA complex in which DNA is wrapped around a histone octamer generally consisting of two copies of each of the four core histones, H2A, H2B, H3 and H4. It is, therefore, not surprising that DNA replication is functionally linked to the structural integrity of chromatin and that defects in the latter can promote replication stress. Moreover, an efficient response to DNA damage associated with stalled or collapsed replication forks requires tightly regulated changes in the surrounding chromatin environment, which may have the potential to alter chromatin at genomic regions prone to replication stress. Finally, the replication of DNA requires the concomitant disruption and reassembly of chromatin. A prolonged disruption due to aberrant replication stalling bears the risk of altering the newly formed chromatin in a way that changes epigenetic information, which may in turn affect gene regulation and/or spatial DNA organization. However, while it is tempting to speculate that such chromatin changes can permanently alter the epigenome, evidence for persistent DNA damage-induced chromatin remodeling is yet to be uncovered. In this review, we will highlight recent advances in our understanding of the role of chromatin during replication stress and discuss possible physiological consequences of replication stress-associated epigenetic defects, which include but are not limited to cellular senescence.

## 2. The Cellular Response to Replication Stress

Before we address the impact of replication (stress) on the epigenetic integrity of our genomes, we will briefly summarize the many factors that can interfere with DNA replication, and how the ensuing replication defects trigger a generally protective replication stress response.

## 3. Sources of Replication Stress

DNA replication relies on the availability and timely synthesis of numerous DNA and chromatin components, including nucleotides, histones and histone chaperones as well as an intact replication machinery. Limited supply of any of these components can be a source of replication stress [4,5]. Additionally, in humans, there are multiple difficult to replicate DNA sequences known as fragile sites, which can be subdivided into late replicating common fragile sites (CFS) and the more recently described early replicating fragile sites (ERFSs) [6,7]. CFSs harbor relatively few active replication origins, making these regions particularly susceptible to DNA polymerase stalling [8]. CFS fragility can further be triggered by the collision of replication and transcription machineries, as a number of CFSs contain large genes, which can take more than one full cell cycle to transcribe [7,9]. RNA/DNA polymerase encounters also appear to be at the heart of ERFS instability, as ERFSs are located in gene rich, highly transcriptionally active parts of the genome [6]. For a comprehensive overview on fragile site biology, we refer the reader to a recent review by Franchitto and Pichierri [7]. At the molecular level, replication stress is frequently associated with stable secondary DNA structures that are distinct from the canonical, right-handed double helix B DNA and present a challenge for the progression of replication forks. Non-B DNA structures include cruciforms, hairpins, triple-stranded H-DNA, left-handed Z-DNA and G-quadruplex (G4) structures, which form in the genome at specific repetitive DNA sequences often associated with fragile DNA elements. A detailed description of sources of DNA replication stress is summarized elsewhere [10].

## 4. Replication Stress and DNA Damage Signaling

Stalled replication forks are a major source of endogenous DNA damage, causing both single-stranded (ss) and double-stranded DNA (dsDNA) lesions when unrepaired. As a result, several pathways are in place to sense and ensure the resolution of stalled replication forks. “Sensing” involves the detection of stretches of ssDNA that form when replicative DNA helicases keep unwinding DNA after DNA polymerase has stalled [4]. SsDNA is recognized and bound by replication protein A (RPA), which triggers the recruitment and activation of several replication-stress response mediators that centrally involve the DDR kinase ataxia telangiectasia mutated and Rad3-related (ATR) [11]. RPA also recruits the RAD9-HUS1-RAD1 (9-1-1) complex to DNA lesions, which regulates ATR by recruiting the allosteric activator topoisomease binding protein 1 (TOPBP1). ATR complex assembly at stalled replication forks signals the cells to coordinate replication, cell cycle and repair. One of the most important downstream targets for ATR kinase is Ser/Thr kinase checkpoint kinase-1 (CHK1), which in turn controls checkpoint signaling via the phosphorylation of cyclin-dependent kinases (CDKs) [11,12,13]. Additionally, ATR signaling plays an important role in inhibiting excessive origin firing [14,15,16]. Highlighting its central role during DNA replication, loss of ATR sensitizes cells to replication stress, causing checkpoint defects and, ultimately chromosome aberrations, which are particularly apparent at fragile sites in both cell lines and mice [6,17,18]. For a detailed overview of ATR function during replication stress, we refer the reader to a comprehensive review by Cimprich and Cortez [11].

Under conditions of persistent replication fork arrest, the cell may not be able to rescue the stalled fork, which can result in DNA double strand break (DSB) formation and a concomitant replication fork collapse. The latter triggers DNA damage signaling via the DSB-sensing ataxia telangiectasia mutated (ATM) kinase [4]. ATM activation promotes the recruitment of the MRE11, RAD51 and NBS1 (MRN) complex to collapsed replication forks, where it facilitates DNA end resection and DSB repair via homologous recombination (HR) [19]. Notably, several studies indicate that ATR signaling is similarly linked to MRN complex recruitment [20,21,22]. In addition to initiating HR, ATM affects replication fork restart by regulating DNA helicases Werner syndrome protein (WRN) and BLM, which are required to resolve replication intermediates [10,23]. The significance of ATM signaling under replication stress is emphasized by the fact that simultaneous loss of ATM and ATR results in increased instability at CFSs as compared to the depletion of ATR alone [24].

## 5. Replication Stress Signaling Drives Senescence

The persistent activation of DNA damage signaling in response to continuous replication stress was found to results in a state of irreversible cell cycle arrest, or cellular senescence [25,26]. Senescence is characterized by significant changes in cell morphology and function, and has been linked to a number of age-related organ pathologies [25]. Notably, senescence can trigger the aberrant secretion of cytokines, a feature termed senescence-associated secretory phenotype (SASP), which has been linked to both chronic inflammatory diseases and cancer [27,28,29,30,31]. A detailed overview of causes and consequences of cellular senescence is discussed elsewhere [25].

A central driver of replication stress-induced senescence in primary cells is the activation of oncogenes, such as tumorigenic mutants of HRAS and BRAF or increased MYC expression [26,32,33]. Oncogene activation results in an initial phase of hyper-proliferation, which triggers excessive DNA replication, multiple origin activation and, eventually, replication stress-associated DDR activation [34,35]. Kinetic studies have shown that the timing for DDR activation coincides with the timing of cells entering senescence, suggesting a role for DDR signaling in the establishment of the latter. Consistent with this, both ATM and ATR signaling are required to initiate OIS [34,35,36]. Remarkably, OIS was recently shown to be established and maintained through an ATM-dependent repression of nucleotide synthesis, further corroborating the link between replication stress, DDR and senescence [37,38]. Together, these findings underline the central role of a tightly regulated cellular response to replication stress, both in maintaining genome integrity and in preventing permanent cell cycle arrest and age-associated functional decline.

## 6. A Role for Chromatin in the Replication Stress Response

Replication fork progression, as well as the resolution of stalled or collapsed forks, occur in a highly organized chromatin environment. In the following, we will discuss (i) how DNA polymerase progression (and stalling) is influenced by the pre-existing chromatin environment; and (ii) how replication stress-induced chromatin changes can affect both the repair of stalled and/or collapsed forks and the epigenetic integrity of the surrounding chromatin environment.

## 7. Impact of Pre-Existing Chromatin on DNA Replication

### 7.1. Repressive Chromatin

Genome-wide as well as locus specific analyses suggest that chromatin accessibility is an important regulator of DNA replication timing. Specifically, less accessible, heterochromatic regions enriched for silent chromatin marks, such as tri-methylated histone H3K9 (H3K9me3), replicate later than more accessible, transcriptionally active euchromatic regions [39]. Consistent with a causal role for H3K9 methylation in replication timing, increased H3K9me3 de-methylation upon overexpression of the KDM4A/JMJD2A demethylase was found to promote chromatin accessibility and, thus, accelerate cell cycle progression as well as replication timing at regions normally enriched for this mark, whereas loss of KDM4A had the opposite effect both in mammalian cells and *C. elegans* [40]. KDM4A-associated changes in DNA replication timing had significant functional consequences: slowed DNA replication due to KDM4A depletion resulted in a replication stress-associated increase in DNA damage and ATR/p53-dependent apoptosis [40]. KDM4A overexpression, on the other hand, caused transient, site-specific copy number gains due to DNA re-replication [41]. Importantly, S phase defects related to KDM4A overexpression could be suppressed by overexpression of the H3K9 methyltransferase Suv39H1 or the H3K9me3-binding protein heterochromatin protein 1-γ (HP1-γ) [40,41]. Altogether, these findings point towards a conserved role of KDM4A and H3K9 methylation in preventing replication stress. This observation may extend to other forms of heterochromatin, as loss of H3K27 monomethylation, a precursor for the polycomb repressive mark H3K27me3, has been linked to severe replication stress in *Tetrahymena* [42]. Moreover, the *D. melanogaster* heterochromatin-associated protein stwl was found to protect from replication stress, presumably by maintaining accurate H3K27 and H3K9 tri-methylation patterns [43]. However, the role for H3K27 methylation in mammalian cells remains to be investigated.

### 7.2. H2B Ubiquitin

The monoubquitination of H2B represents another histone modification that has been reported to cause replication stress when perturbed. In yeast, H2Bub1 at lysine 123 has been mapped to chromatin surrounding replication origins, where it facilitates the assembly or stability of newly synthesized nucleosomes following DNA replication. Consistent with this, loss of H2Bub1 slows replication fork progression without affecting the assembly of the pre-replication complex [44]. As a result, yeast cells with mutated H2B-K123 are hypersensitive to replication stress induced by hydroxyurea (HU), an inhibitor of dNTP synthesis [45], and show slow recovery of DNA replication after removal of the HU block. Although the precise molecular mechanism for H2Bub1 function remains to be determined, H2B monoubiquitination appears to be a critical aspect of replisome stability [44]. The effect of H2B ubiquitination on nucleosome assembly/stability is in striking similarity to its role during transcription, where H2Bub1 promotes RNA polymerase progression and, hence, transcript elongation [46]. Given that the latter is conserved in human cells, it is tempting to speculate that the same holds true for the control of DNA replication by H2Bub1. Consistent with this notion, depletion of RNF20/40, the mammalian ortholog of the yeast H2B E3 ligases BRE1A/B, causes replication stress and genomic instability [47].

## 8. Replication Stress-Associated Chromatin Reorganization

DNA replication is not only influenced by chromatin, but can significantly alter the latter [48]. Replication stress triggers a cellular response to DNA damage following the formation of ssDNA, and ultimately DSBs, at stalled or collapsed replication forks, respectively. It is, therefore, not surprising that several of the chromatin changes implicated in DNA repair have now been linked to replication stress. A list of replication stress-associated chromatin modifiers and modifications is provided in Table 1. In the following, we will highlight some of the most pronounced effects on replication fork-surrounding chromatin, particularly those that may contribute to (persistent) epigenetic deregulation and concomitant changes in cell function.

**Table 1:** Chromatin modifications and modifiers involved in replication stress (RS). Relevant in mammalian cells unless noted otherwise.

Chromatin Component	Modifiers/Interactors	Major Functions during RS	References
**Histone modifications**			
γ-H2AX (phospho-S139) γ-H2A (phospho-S129)	ATM/ATR Mec1/Tel1 (yeast)	Replication fork progression, repair of collapsed forks.	[49,50,51,52]
H3 phospho-T45	Cdc-DbF4 (yeast)	Increases resistance to RS.	[53]
H3K56ac	HAT: RTT109 (yeast) HDAC: Hst3/4 (yeast)	Pre-disposition mark on nascent chromatin, promotes replication fork stability.	[54,55]
H4K5ac, H4K12ac	HAT: HAT1 HDAC: HDAC1-3	Pre-disposition mark on nascent chromatin, promotes replication fork stability.	[56,57,58]
H3K4me3	HMT: Set1p (yeast) HMT: METNASE	Facilitates DSB repair at collapsed forks. Facilitates replication fork restart.	[59,60]
H3K9me	HMT:Clr4 (yeast) HMT: SUV39H1 KDM: KDM4A	Promotes HU induced S/M checkpoint. Controls replication timing and replication fork stability.	[40,41,61]
H3K36me3	HMT: Set2p (yeast)	Promotes HU induced S/M checkpoint. Facilitates replication fork restart.	[61]
H3K27me1	HMT: TXR1 (tetrahymena)	Protects from RS.	[42]
H3K79me3	Dot1 (yeast)	Promotes sister chromatid recombination after RS.	[62]
H4K20me	HMT: WHSC1	Identified in genetic screen for genes involved in RS.	[63]
H2Aub1	DUB: USP3	Facilitates DSB repair at collapsed forks.	[64]
H2Bub1	Bre1 (yeast)	Nucleosome re-assembly, replisome stability	[44]
Poly(ADP-)ribose	PARP/PARG	Modulates RPA accumulation at collapsed forks.	[65]
**Chaperones and remodelers**			
ASF1	H3/H4	H3/H4 chaperone, promotes nucleosome reassembly at sites of RS.	[48]
ATRX	Histone 3.3	H3.3 chaperone, promotes nucleosome reassembly, fork progression.	[66,67]
INO80	H2A.Z (yeast)	Removes H2A.Z from nucleosomes, promotes recovery of stalled forks.	[68,69,70]
RSC2/BAF180	PCNA	Promotes recovery of stalled replication forks.	[71]
MMS22L	TONSL ASF1, FACT	Promotes recovery from RS, facilitates RAD51 loading and HR. Nucleosome reassembly	[72,73,74,75,76]
NASP	H3/H4	H3/H4 chaperone, regulates histone metabolism during RS.	[77]
SAFB1	Chromatin	Scaffold protein, regulates γ-H2AX-spreading during RS.	[78]

HAT: histone acetyltransferase; HDAC: histone deacetylase; HMT: histone methyltransferase; KDM: lysine demethylase.

### 8.1. γ-H2AX

One of the first chromatin changes in response to DNA damage is the phosphorylation of histone H2AX on S139 (γ-H2AX). Genome-wide mapping of the yeast γ-H2AX homolog γ-H2A by chromatin immunoprecipitation (ChIP) demonstrated that γ-H2A is enriched at sites of replication fork stalling in S phase, which are predominantly localized to ribosomal DNA (rDNA) and tRNA genes, telomeres and actively repressed protein coding genes [51]. While γ-H2AX is often considered a marker for DSBs, accumulating evidence points to a more general role during replication stress, implicating γ-H2AX both in replication fork stalling in the absence of DSBs and in the repair of collapsed replication forks (DSBs). Supporting a DSB-independent role, γ-H2AX can form foci at sites of replication stress in an ATR-dependent manner [79]. Moreover, using the recently developed isolation of Proteins On Nascent DNA (iPOND), it has been demonstrated that ATR-dependent γ-H2AX formation at stalled forks occurs within 10 min of HU treatment, well before the accumulation of the DSB markers Mre11, DNA-PKs and Ku70/Ku80 [49]. Persistent accumulation of γ-H2AX at collapsed forks, on the other hand, is dependent on ATM, consistent with its function in DSB repair [49]. Distinct roles for ATM and ATR in the phosphorylation of H2AX upon replication stress have been corroborated by several studies [6,79,80]. Notably, combined deficiency for ATR and γ-H2AX results in increased genomic instability under replication stress [50]. A similar effect was observed in ATR deficient cells that lack ATM [24], underlining the synergistic roles of ATM and ATR with regard to replication stress-induced H2AX phosphorylation and the subsequent initiation of DNA repair.

Recently, it has been demonstrated that the chromatin-associated scaffold attachment factor SAFB1 regulates γ-H2AX spreading during genotoxic stress signaling [78]. Importantly, SAFB1 is required for replication fork stability and cell-cycle checkpoint induction, indicating that accurate control of γ-H2AX formation is a critical aspect for genome maintenance during replication stress. Consistent with this, genome-wide mapping studies of γ-H2AX spreading around DSBs suggest that γ-H2AX propagates in a tightly regulated manner, being excluded from active genes in order for transcription to be accurately maintained within γ-H2AX domains [81]. Altogether, the DDR-dependent phosphorylation of H2AX underlines the functional similarities between DSB repair and the cellular response to replication stress. Future work will no doubt reveal many other DSB-associated chromatin changes to be implicated in the repair of collapsed replication forks, particularly those with known roles in HR. It will be interesting to determine their (lasting?) impact on the epigenetic integrity of difficult to replicate chromatin domains.

### 8.2. Chromatin Remodeling

**Figure 1:**
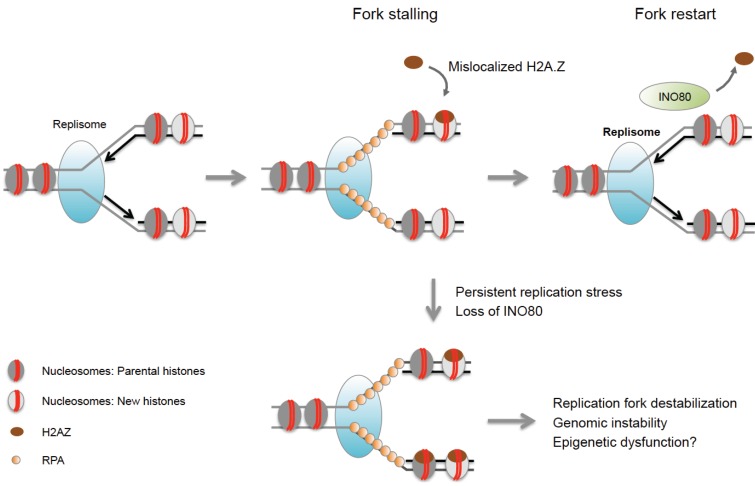
INO80 stabilizes replication forks and counteracts mislocalization of H2A.Z. Excess H2A.Z can cause replication defects and genome instability in the absence of the INO80 chromatin remodeler, which facilitates the removal of H2A.Z from chromatin. Aberrant H2A.Z accumulation may alter the epigenetic landscape at sites of replication stress.

In addition to histone modifications, chromatin remodeling and histone variant exchange have been associated with the response to replication stress in both yeast and mammalian cells. Depletion of the chromatin remodeler INO80 results in impaired fork progression, destabilization and collapse of replication forks and reduced cell viability in yeast [68,70]. Interestingly, mammalian cells with defects in INO80 exhibit increased sensitivity to HU, suggesting a conserved role for this enzyme during replication stress [82,83]. Although its precise function remains to be investigated, INO80 was shown to promote the removal of mislocalized H2A.Z, and replication defects in INO80 mutants can, in turn, be rescued by reducing endogenous H2A.Z levels [69]. Excessive H2A.Z incorporation is thought to trigger fork collapse via poorly understood mechanisms [69], suggesting that INO80 may act by stabilizing stalled replication forks (Figure 1). Consistent with an unfavorable role for H2A.Z at stalled forks, this variant is underrepresented at replication forks even in the absence of replication stress [84,85]. Notably, differential incorporation of histone variants at replication forks is not limited to H2A.Z, but extends to other variants including macroH2A2 and H2A.Bbd [85,86]. While macro-histones have not (yet) been implicated in replication stress, H2A.Bbd was found to be transiently enriched at sites of DNA synthesis during S phase [86]. Aberrant expression or loss of macro-histones and the H2A.Bbd variant has further been linked to DNA damage susceptibility during S phase and/or HR defects [86,87]. It will, thus, be of particular interest to determine if and how these variants may affect chromatin integrity under conditions of replication stress.

Another ATP-dependent chromatin-remodeling factor, α thalassaemia/mental retardation X-linked (ATRX), was recently shown to play an important role in replication stress tolerance. ATRX deficient cells are associated with replication defects such as an increase in replication fork stalling and a delayed progression through S phase [66]. Notably, ATRX mediates the selective incorporation of the histone variant H3.3 [67], which may account for accurate H3.3 maintenance at replication forks and thus at least in part explain replication stress in the absence of ATRX. ATRX was found to bind G4-structured DNA, suggesting that its role during replication stress is most prominent at DNA elements with G-rich tandem repeats. Indeed, increased γ-H2AX accumulation in ATRX deficient cells was most notable at telomeric DNA, a known site of G4 formation [88].

### 8.3. Histone Acetylation and Nucleosome Reassembly

Both DNA repair and DNA replication rely on the incorporation of (newly synthesized) histones to re-establish a functional chromatin environment at sites of nucleosome depletion, and several histone remodelers and modifiers have been implicated in this process. So-called pre-disposition marks facilitate the transfer of newly synthesized histone H3/H4 dimers to the nuclear chromatin assembly factor CAF1 via ASF1 and other chaperones [48]. The most highly conserved pre-disposition mark is the di-acetylation of H4 at K5 and K12, which requires the H4K5K12 lysine acetyltransferase RBAP46/HAT1 [56]. Histone H3 is also acetylated before deposition onto DNA, but preferential sites of acetylation vary between species, including H3K56ac and H3K27ac in budding yeast, and H3K14ac and H3K18ac in human cells [48]. Acetylation of nascent chromatin helps attenuate H1 deposition to prevent higher order compaction during the replication process [89]. The proper assembly and maturation of nascent chromatin is, in turn, essential for replication fork progression and stability. Defects in H3K56Ac-mediated chromatin reassembly cause replication fork collapse, hyper-recombination and large chromosomal rearrangements in yeast [54,90,91]. Notably, the subsequent deacetylation of pre-disposition marks via class I histone deacetylases HDAC1, 2 and 3 as well as certain class III HDACs (sirtuins) appears to be equally important for replication fork progression and/or restart [57,58,92]. Consistent with the latter, treatment with HDAC inhibitors as well as HDAC3 depletion can slow fork speed in human cells, resulting in S phase DNA damage, chromosome fragility and, when deleted specifically in the liver, development of hepatocellular carcinoma [1,58,93]. Moreover, a failure to remove pre-disposition acetyl-marks was found to promote aberrant chromatin de-condensation of pericentric heterochromatin, causing severe chromosome segregation defects [94].

Several recent observations further underline the complexity of tightly controlled nucleosome reassembly and its importance for accurate DNA replication. Mutations in codanin, a novel ASF1-interacting partner linked to congenital dyserythropoietic anaemia type I (CDAI), are characterized by chromatin abnormalities and replication defects [95]. Moreover, ASF1 was recently found to interact with the DSB repair protein MMS22 like protein (MMS22L), which further associated with the FACT chromatin remodeling complex as well as histones, suggesting that it may be functionally linked to nucleosome assembly/disassembly [75,76]. Supporting a direct role in replication stress, MMS22L forms a complex with the leucine-rich TONSL/NFKBIL2 protein at damaged forks to regulate HR by promoting RAD51 loading via RPA displacement [72,73,74,75]. This function is likely to be evolutionarily conserved as both yeast mms2p and the *A. thaliana* TONSL ortholog BRU1 are required for replisome integrity [76,96]. It will be interesting to determine if and how the repair activity of MMS22L is related to its potential role in nucleosome remodeling.

**Figure 2:**
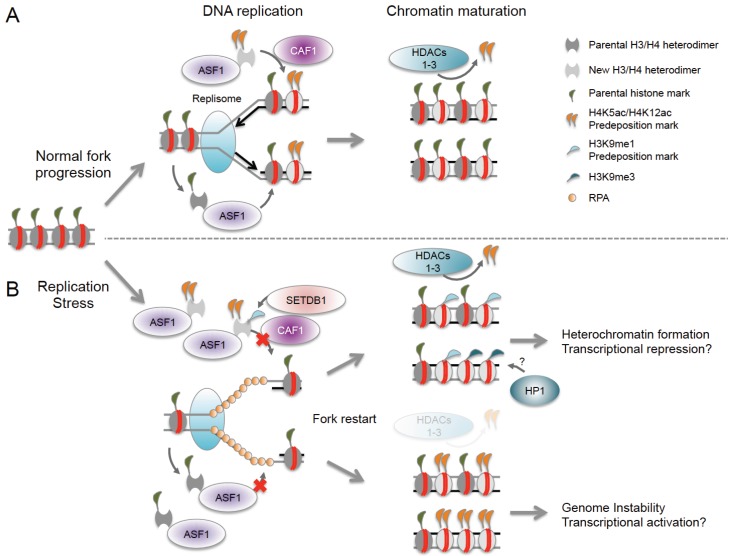
Epigenetic consequences of nucleosome reassembly defects at stalled replication forks. (**A**) Reassembly of histone H3/H4 heterodimers at replication forks is mediated by the sequential actions of the chromatin assembly factors ASF1 and CAF1. New histones carry H4K5ac/H4K12ac predisposition marks, which are required for their nuclear import. ASF1 can bind both new and evicted histones, ensuring tightly controlled histone recycling and nucleosome reassembly; (**B**) Replication stress interferes with the restoration of epigenetic information at stalled forks. Fork arrest results in excessive histone eviction and accumulation of ASF1 loaded with both old and new histones. Fork restart triggers rapid nucleosome assembly, which can result in unbalanced incorporation of old and new histones. Replication stress also promotes the accumulation of the H3K9me1 pre-deposition mark, which may serve as a template for H3K9me3 and HP1-mediated heterochromatin formation. Finally, impaired HDAC function may affect the removal of pre-deposition acetyl-marks, causing epigenetic changes in post-replication chromatin. Together, these defects have the potential to either promote or inhibit the expression of nearby genes.

Notably, ASF1 associates not only with newly synthesized but also with evicted histones and can act as a buffer for excess histones under conditions of replication stress [48,97]. Upon replication fork restart, ASF1 facilitates the rapid, but possibly imbalanced incorporation of new and old histones, which can interfere with otherwise tightly orchestrated predisposition marking and histone recycling (Figure 2). Replication stress was further found to increase the H3K9me1 predisposition mark on available ASF1-bound H3, which may prime nascent chromatin for the formation of aberrant H3K9me3 domains [97,98]. It is tempting to speculate that the accumulation of these repressive marks may account for unscheduled heterochromatinization at sites of natural fork stalling, such as fragile DNA elements. Aberrant histone deposition has also been observed at post-replicative gaps that form due to replication re-initiation downstream of an arrested fork, and histone deposition at these sites becomes uncoupled from the normal replication-associated recycling of evicted histones. The latter can lead to the deposition of naive histones lacking the parental marks associated with these regions, which can in turn alter epigenetic integrity beyond the sites of replication stress [48,99].

## 9. Physiological Consequences of Replication Stress-Induced Chromatin Reorganization

Changes in chromatin structure can cause numerous defects in cell function as a result of altered nuclear organization, deregulation of gene expression and/or increased genome instability. Consequently, epigenetic dysfunction has been linked to age-associated degenerative diseases as well as cancer formation or progression [100,101]. Having established replication stress as a driver of chromatin reorganization, we will now discuss its potential impact on epigenetic integrity and cell function.

## 10. Replication Stress and Aberrant Gene Expression

Several lines of evidence suggest that replication stress-induced epigenetic changes can alter gene expression. Recently, it has been demonstrated that replication stress caused by the DNA intercalator doxorubicin can promote ATR-dependent transcriptional repression of genes in close vicinity to stalled forks by modulating nucleosome reassembly [102]. In addition, depletion of deoxyribonucleotides, which is a major cause of replication stress, was found to promote aberrant spreading of repressive H3K9me3 marks at mating type loci in fission yeast, suggesting a link between the two processes [103]. Consistent with this notion, replication stalling was found to promote unscheduled gene silencing in budding yeast by facilitating chromatin binding of the silent information regulator complex (SIR), which is required for the repression of mating type loci, telomeres and ribosomal DNA and facilitates H3K9 methylation in fission yeast [104,105]. Whether a similar phenomenon takes place in mammals remains to be investigated. However, the observation that the mammalian SIR2 ortholog SIRT1 is redistributed on chromatin upon DNA damage may suggest a role for sirtuins in replication stress-induced gene deregulation in higher organisms [106].

Replication fork stalling due to structural obstacles in DNA can also cause perturbations in local gene expression. The latter may be attributed to the uncoupling of DNA replication and the recycling of parental histones (see above), which will in turn alter the propagation of histone marks during chromosomal replication (Figure 2B). One example is the interruption of processive DNA replication at unresolved G-quadruplex sequences, which was shown to lead to a biased incorporation of newly synthesized, H4-acetylated histones and a concomitant loss of parental repressive chromatin modifications in chicken DT40 cells [99]. Notably, G4 structures may also cause replication stress-induced gene repression in a process involving ATRX-mediated chromatin remodeling. ATRX binds to G4-forming variable number tandem repeat regions (VNTRs) near the α-globin gene cluster, and genes in in this region become repressed in ATRX mutant cells [107]. Consistent with this, ATRX was found to modulate gene expression by facilitating the incorporation of histone H3.3, which is often found in transcriptionally active chromatin [67,108]. However, defective incorporation of H3.3 at sites of ATRX-associated G4 structures remains to be demonstrated [107]. Interestingly, G4-associated replication stress was recently shown to be specific to leading-strand replication in yeast, raising the intriguing possibility of asymmetric chromatin changes at G4 domains [109]. Epigenetic differences between sister chromatids have, in turn, been suggested to contribute to asymmetric cell division in stem cells, pointing to a potential role for replication stress in this process [110]. In analogy to G4 structures, triplet-repeat expansions can form secondary DNA structures that interfere with replication fork progression and were found to promote the silencing of a nearby reporter gene [111]. The latter involves HP1-sensitive position effect variegation, and it will be interesting to determine if this process is directly affected by the replication stress response. Replication stress-induced gene silencing is consistent with the notion that replication fork stalling or collapse can promote aberrant heterochromatin formation due to unscheduled H3K9me1 incorporation and subsequent H3K9 trimethylation (Figure 2B, [97]). It remains to be determined if these and other replication stress-induced chromatin changes, including the deregulated deposition of H3.3 and/or H2A.Z variants (see Figure 1), can permanently alter the epigenetic landscape of dividing cells, thereby altering transcription profiles and, ultimately, cell function.

## 11. Replication Stress Induced Chromatin Reorganization in OIS

In addition to local gene deregulation, replication stress has been implicated in a more global change in chromatin structure during oncogene-induced cellular senescence [26]. The latter results in the condensation of one or more single chromosomes to form so-called senescence-associated heterochromatin foci (SAHF), which are enriched in common markers of constitutive heterochromatin, including hypoacetylated histones, H3K9 methylation, macro-histone variants and HP1 proteins [36,112,113]. Notably, SAHFs are formed preferentially in response to OIS, whereas replicative senescence can occur in their absence. This notion is consistent with the observation that repressive chromatin, and specifically H3K9 methylation, is required to maintain attenuated DNA damage signaling in response to oncogene-induced replication stress, which promotes cell cycle arrest in the absence of apoptosis as a barrier to malignant transformation [36]. Although the molecular mechanisms involved in SAHF formation are not well understood, several proteins have been shown to contribute to this process, including the histone chaperones HIRA and ASF1, as well as HP1-γ, HMGA proteins and the H3K9 tri-methyltransferases SUV39H1/H2 [36,113,114,115]. The fact that both the enrichment of H3K9me3 and the formation of SAHFs are ATR-dependent suggests a direct link to replication stress [36]. Interestingly, SUV39H1 was found to be enriched at nascent chromatin near replication forks [84]. It will, thus, be of interest to determine if and how H3K9 methylation is altered during replicative senescence, *i.e.*, in the absence of oncogenic stress, and whether attenuated DNA damage signaling can promote senescence under these conditions. Notably, both OIS and replicative senescence have been linked to genome-wide changes in the distribution of the repressive H3K27me3 mark, and changes in H3K27 methylation are associated with replication stress in lower organisms, suggesting a more general role for repressive chromatin during both processes [2,42]. However, if the reorganization of repressive chromatin observed during replicative senescence is a cause of the senescence process or rather a protective response gone awry remains to be determined.

## 12. Conclusions and Perspective

Replication stress is a major source of endogenous DNA damage. As a result, cells have developed a sophisticated response to deal with the many challenges to DNA polymerase progression and, thereby ensure accurate DNA replication across the genome. Once polymerase is stalled, however, the surrounding chromatin environment is inevitably exposed to numerous epigenetic changes. While this review highlights recent advances in our understanding of replication stress-induced chromatin reorganization, many questions remain: What regulates the accurate restoration of replication stress-associated chromatin? Can replication stress-induced chromatin changes persist and thereby alter the epigenetic landscape and/or differentiation state of a cell? Are such changes at least in part responsible for the epigenetic dysfunction observed with age and/or during cellular senescence? Can we manipulate the cellular response to replication stress through chromatin? Finally, it is worth noting that changes in chromatin structure may not merely be a consequence of replication stress. Cellular processes that alter chromatin states, such as differentiation or stress responses, may indeed act to *cause* replication stress, as both replication timing and DNA polymerase progression are tightly linked to the surrounding chromatin environment [116]. Taken together, evidence is accumulating that, while the factors that mediate the repair of stalled and collapsed replication forks are critical to ensure genome maintenance, a possibly equally essential role may be played by those factors that reorganize, modify and reassemble fork-associated chromatin to ensure accurate maintenance of epigenetic information.
